# Supplementary material for: Identification of photoperiod-induced specific miRNAs in the adrenal glands of Sunite sheep (Ovis aries)
Source: Front Vet Sci. 2022 Jul 22;9:888207. doi: 10.3389/fvets.2022.888207 (PMC9354845; doi:10.3389/fvets.2022.888207)
Supplement: Supplementary file 1 [file Data_Sheet_1.ZIP › Supplementary file/miRDeep2 file.zip!/miRDeep2 file/Novel_Structure/SPLP42A3/Novel_82.pdf]

Provisional ID	:	NC_019468.2_20177
Score total	:	1.4
Score for star read(s)	:	-1.3
Score for read counts	:	0
Score for mfe	:	1.1
Score for randfold	:	1.6
Score for cons. seed	:	
Total read count	:	64
Mature read count	:	49
Loop read count	:	0
Star read count	:	15

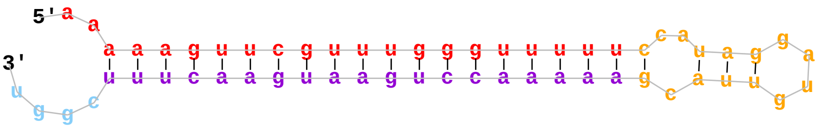

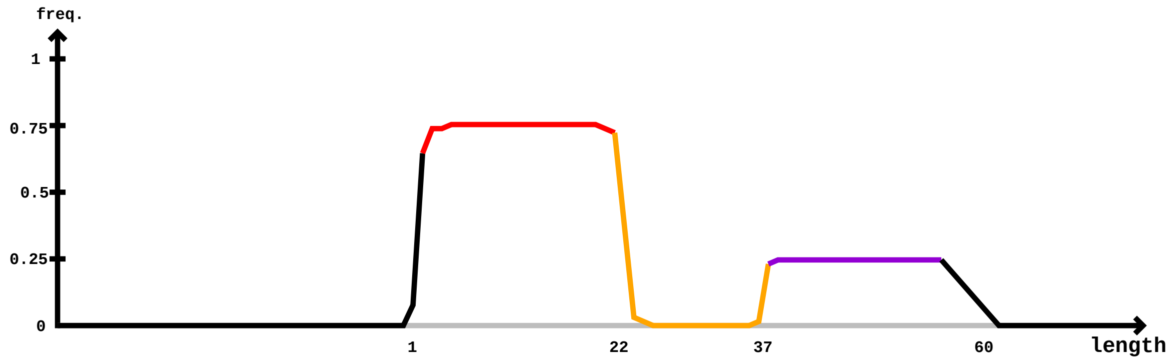

Mature   Star

```
5'- gaaaccauaguaauuagaaauguuacuggcuuggccaaaaaguucguuuggguuuuuccauaggauguuacgaaaaaccugaaugaacuuucgguucaacccagaagaaug  -3'   obs
    gaaaccauaguaauuagaaauguuacuggcuuggccaaaaaguucguuuggguuuuuccauaggauguuacgaaaaaccugaaugaacuuucgguucaacccagaagaaug         exp
    .........................((((.((((((..((((((((((((((((((((..(((....))).)))))))))))))))))))).)).)))).)))).......   reads   mm   sample
    ...................................caaaaaguucguuuggguuC........................................................   1       1    seq
    ...................................caaaaaguucguuCggguuuu.......................................................   1       1    seq
    ...................................caaaaaguucguuCggguuuuu......................................................   3       1    seq
    ....................................aaaaaguucguuCggguuuuu......................................................   5       1    seq
    ....................................aaaaaguucguuuggguuuuG......................................................   1       1    seq
    ....................................aaaaaguucguuuggguuuuC......................................................   2       1    seq
    ....................................aaaaaguucguuuggguuuuu......................................................   24      0    seq
    ....................................aaaaaguuUguuuggguuuuu......................................................   1       1    seq
    ....................................aaaaaguucguuuggguuCuuc.....................................................   1       1    seq
    ....................................aaaaaguuUguuuggguuuuuc.....................................................   2       1    seq
    ....................................aaaaaguucguuuggguuuuuA.....................................................   1       1    seq
    .....................................aaaaguucguuuggguuuuuA.....................................................   5       1    seq
    .....................................aaaaguucguuuggguuuuucAa...................................................   1       1    seq
    .......................................aaguucAuuuggguuuuucc....................................................   1       1    seq
    .......................................................................gaaaaaccCgaaugaacuuu....................   1       1    seq
    ........................................................................aaaaaccugaaugaacCuu....................   1       1    seq
    ........................................................................aaaaaccCgaaugaacuuu....................   1       1    seq
    ........................................................................aaaaaUcugaaugaacuuu....................   12      1    seq
    .........................................................................aaaacUugaaugaacuuuc...................   1       1    seq
```
